# 

**Published:** 1894-03-10

**Authors:** 


### CONTENTS.

**Insanity and its Kinships** - - - - - - - - - - 407

**Around the Hospitals** - - - - - - - - - - - 408

**On Modern Progress in Ophthalmic Medicine and Su gery.** By ROBERT BRUDENELL CARTER, F.R.C.S. - - 409

**Art and Disease.** VII.—Aspects of Death - - - - - - 410

**Drugs and Their Uses** - - - - - - - - - 411

**The Field of Science** - - - - - - - - - - 412

**Annotations.**—The Doctor or the Chemist?—"Selection" and Race Progress—Should Journalists Marry? - - - - - 413

**Notes and News** - - - - - - - - - - - 414

**Scraps and Gleanings** - - - - - - - - - - 426

**The Book World of Medicine and Science** - - - - 427

**Medical Progress and Hospital Clinics—**

On the Treatment of Rupture of Urethra - - - - - 415

Empyema of the Antrum - - - - - - - - 416

MEDICAL PROGRESS.—Nerve Diseases - - - - - - 417

Tubercular Peritonitis, &c. - - - - - - - 419

Diseases of the Tonsils - - - - - - - - 420

NEW DRUGS AND PREPARATIONS - - - - - - - 421

NEW APPLIANCES AND THINGS MEDICAL - - - - - 422

**The Institutional Workshop—**

Bristol General Hospital - - - - - - - - 423

THE STORY OF THE INSANE FROM YEAR TO YEAR - - - 424

HOSPITAL FESTIVALS, MEETINGS, &c. - - - - - - 425

### EXTRA SUPPLEMENT.—THE NURSING MIRROR.

**News from the Nursing World.**—A Royal Stall-holder—Openings for Women—The East London Nursing Society—North London Nursing Association—Characters and Co-operators—Lady Nurses—Unique Training—Trouble at Macclesfield—Wise Scotch Lassies—District Nursing in Ireland—Instruction and Entertainment—Testimonials—Kindly Inspection—Our Correspondents—Short Items - - ccxxi
**On General Nursing.** II.—Measles (*continued*) - - - - ccxxiii
**Massage in Java** - - - - - - - - - - ccxxiv
**Nursing in New York.**—The Tenement District of the City ccxxv
**Lecture on Diphtheria** - - - - - - - - - ccxxv
**Can Medical Women Nurse?** - - - - - - - - ccxxv
**Women's Work.**—Women Librarians - - - - - - ccxxvi
**Madame Patey—Notes and Queries** - - - - - ccxxvi
**Our Examinations** - - - - - - - - - - ccxxvii
**Presentations—Minor Appointments** - - - - - ccxxvii
**For Reading to the Sick** - - - - - - - - ccxxviii
**The Muses' Looking Glass.**—A Groat's Worth of Wit - ccxxix
**Everybody's Opinion** - - - - - - - - - ccxxx
**Where to Go?—Appointments** - - - - - - - ccxxx

